# Embracing Personalized Strategies in Radiotherapy for Nasopharyngeal Carcinoma: Beyond the Conventional Bounds of Fields and Borders

**DOI:** 10.3390/cancers16020383

**Published:** 2024-01-16

**Authors:** Pui Lam Yip, Rui You, Ming-Yuan Chen, Melvin L. K. Chua

**Affiliations:** 1 Department of Radiation Oncology, National University Cancer Institute, National University Hospital, Singapore 119074, Singapore; jessie_yip@nuhs.edu.sg; 2 Department of Nasopharyngeal Carcinoma, Sun Yat-sen University Cancer Center, Guangzhou 510060, China; yourui@sysucc.org.cn (R.Y.); chenmy@sysucc.org.cn (M.-Y.C.); 3 State Key Laboratory of Oncology in South China, Collaborative Innovation Center for Cancer Medicine, Sun Yat-sen University Cancer Center, Guangzhou 510060, China; 4 Guangdong Key Laboratory of Nasopharyngeal Carcinoma Diagnosis and Therapy, Guangzhou 510060, China; 5 Cooperative Surgical Ward of Nasopharyngeal Carcinoma, Faifth Affiliated Hospital of Guangzhou Medical University, Guangzhou 510700, China; 6 Division of Medical Sciences, National Cancer Centre Singapore, 30 Hospital Blvd, Singapore 168583, Singapore; 7 Division of Radiation Oncology, National Cancer Centre Singapore, 30 Hospital Blvd, Singapore 168583, Singapore; 8 Oncology Academic Clinical Programme, Duke-NUS Medical School, Singapore 169857, Singapore

**Keywords:** nasopharyngeal carcinoma, radiotherapy, treatment individualization, response adaptation, toxicity

## Abstract

**Simple Summary:**

In the dynamic landscape of nasopharyngeal carcinoma (NPC) management, this review delves into a spectrum of radiotherapy (RT) practices, ranging from meticulous target delineation to dose prescription and sophisticated treatment delivery. The paper accentuates the evolving landscape of tailored treatment strategies and the departure from conventional one-size-fits-all RT treatment. Noteworthy initiatives, such as volume-reduced RT and adaptive planning, in conjunction with the integration of biomarkers and functional imaging for risk stratification, are highlighted. These efforts aim to inform treatment intensification or de-escalation, advocating for a paradigm shift towards patient-centric NPC management. The research momentum exemplifies a collective commitment to advancing NPC treatment and improving the quality of life in NPC survivors.

**Abstract:**

Radiotherapy is the primary treatment modality for non-metastatic nasopharyngeal carcinoma (NPC) across all TN-stages. Locoregional control rates have been impressive even from the 2D radiotherapy (RT) era, except when the ability to deliver optimal dose coverage to the tumor is compromised. However, short- and long-term complications following head and neck RT are potentially debilitating, and thus, there has been much research investigating technological advances in RT delivery over the past decades, with the primary goal of limiting normal tissue damage. On this note, with a plateau in gains of therapeutic ratio by modern RT techniques, future advances have to be focused on individualization of RT, both in terms of dose prescription and the delineation of target volumes. In this review, we analyzed the guidelines and evidence related to contouring methods, and dose prescription for early and locoregionally advanced (LA-) NPC. Next, with the preference for induction chemotherapy (IC) in patients with LA-NPC, we assessed the evidence concerning radiotherapy adaptations guided by IC response, as well as functional imaging and contour changes during treatment. Finally, we discussed on RT individualization that is guided by EBV DNA assessment, and its importance in the era of combinatorial immune checkpoint blockade therapy with RT.

## 1. Introduction

### An Overview of Nasopharyngeal Carcinoma (NPC) in the Modern Era

Nasopharyngeal carcinoma (NPC) is a distinctive malignancy prevalent in southeast Asia, south-central Asia, and north and east Africa [1]. The World Health Organization (WHO) classification categorizes NPC into keratinizing and non-keratinizing subtypes, and basaloid squamous cell carcinoma (SCC). Notably, the non-keratinizing undifferentiated NPC, formerly referred to as WHO type III, predominates in endemic regions [2]. Endemic NPCs exhibit a favorable prognosis and are considered responsive to both radiation and chemotherapy. A small subset of NPC is associated with human papillomavirus (HPV), which has been observed in both endemic and non-endemic regions. Nevertheless, the clinical outcome and optimal treatment strategies of this subset remain less well-defined [3,4,5].

Much of the recent change in treatment paradigm centered around optimizing systemic therapy. First, concurrent chemoradiation (CCRT) has proven efficacy since the 2000s [6]. Second, induction chemotherapy (IC) followed by CCRT has become the standard of care for locally advanced NPC since the 2020s [7,8,9,10,11,12]. Recently, there has been a resurgence of evidence on adjuvant chemotherapy after three positive trials [13,14,15].

Radiotherapy (RT) remains the cornerstone of NPC treatment. Over the past three decades, RT techniques have undergone substantial development. The use of conventional two-dimensional (2D) RT was the standard in the 1980s. During the 2D era, RT fields typically consisted of multi-phases with shrinking fields. Optic chiasm, brainstem and spinal cord shielding was required at some point during the treatment [16,17]. With the advancement of RT techniques, a transition to a 3D conformal technique transpired, followed by the adoption of intensity-modulated radiotherapy (IMRT) in the 2000s. IMRT has substantially facilitated the precise delivery of conformal radiation doses to the target volume while minimizing radiation exposure to surrounding organs at risk (OARs). The utilization of IMRT has demonstrated associations with reduced xerostomia, temporal lobe neuropathy, and improved overall survival (OS) [18,19]. Compared to older, less conformal RT techniques, IMRT demands precise target volume delineation and treatment delivery to achieve optimal disease control. To standardize and ensure the quality of RT treatment, various guidelines have been published to offer recommendations on target delineation and prescription doses. Continual advancements in treatment have led to improved outcomes in NPC. In a population-based study, the 8-year actuarial overall survival (OS) was over 80% and 50% for early and locally advanced NPC, respectively in the IMRT era. Distant metastasis remains the major failure pattern [20]. Impressively, the 3-year locoregional failure-free survival rates range from 91% to 94% in contemporary cohorts [9,10,21,22].

However, we must not overlook the spectrum of short-term and long-term treatment toxicities. Cisplatin commonly associates with hematological and non-hematological toxicities such as nephropathy, neuropathy, and ototoxicity, which can be persistent after treatment [23,24]. Similarly, high-dose RT gives rise to acute toxicities such as dermatitis, mucositis, weight loss, and dysphagia. Failure to manage severe acute toxicities could lead to fatal outcomes. More importantly, NPC survivors are commonly confronted with long-term toxicities ranged from xerostomia, dental issues, neck fibrosis, dysphagia, hearing impairment, stroke, hypothyroidism, lower cranial nerve palsies, osteoradionecrosis, and temporal lobe necrosis [25,26,27,28,29,30,31].

There is a growing belief that a subset of NPC patients might be subjected to overtreatment under the current treatment paradigm. This is based on the observation that a subset of patients responded more favorably to IC and RT in terms of tumor shrinkage and clearance of tumor markers, which consistently signify better prognosis, and that a group of patients was cured with RT alone. Consequently, a recent initiative has emerged, focusing on risk stratification and personalized treatment intensification or de-intensification with the aim of improving treatment outcomes and reducing acute and late toxicities.

Technological advancements have provided avenues for treatment personalization. First, high-definition MRI, positron emission tomography (PET)/computed tomography (CT), and functional imaging have enhanced diagnosis, staging and target delineation. Second, adaptive RT has become more available and practical. Third, a wealth of knowledge has accumulated around plasma Epstein–Barr virus DNA (EBV DNA) as a dynamic biomarker for treatment adaptation. Lastly, there is the prospect of integrating immune checkpoint inhibitor with RT. On the other hand, successful treatment personalization requires clinicians to be well-versed in real-world practice variations and the relevant scientific evidence, allowing them to tailor treatments to individual patient circumstances. This review will concentrate on these pivotal areas of ongoing research (Table 1).

## 2. Redefining Target Volumes

NPC poses a challenge in RT due to its infiltrative nature and frequent skull base invasion. Several interconnected skull base foramina serve as conduits for tumor spread [32,33]. Accurate contouring necessitates a precise understanding of diagnostic imaging and the intricacies of tumor spread patterns. Studies on locoregional failure patterns revealed that infield failure is predominant [34,35,36,37]. A retrospective analysis of 1039 patients revealed that 7.2% experienced local and/or regional recurrences, with 88% being in-field failures (defined as at least 95% of the recurrent tumor locating within the 95% isodose). In addition, they found that infield local failure was associated with a primary tumor volume exceeding 68.8 mL and the histological subtype of non-keratinizing differentiated carcinoma. On the other hand, infield nodal failure correlated with a nodal volume of 19.9 mL and cervical nodal necrosis [37]. Consequently, two implications arise: firstly, current contouring seems to provide adequate coverage, indicating a potential for volume reduction, and secondly, further investigation is required to comprehend radio-resistance and explore potential dose escalation to reduce infield failure.

### 2.1. Contrasting the International Consensus Guideline and the Chinese Protocol

There are two prominent protocols regarding contouring methods. An international guideline [38], published in 2017, delineates the clinical target volumes of the primary (CTV_p_) with two distinct dose levels: (1) a high-dose CTV (approximately 70 Gy equivalent) covering the gross tumor volume in nasopharynx (GTV_NP_) with a 5 mm margin, and (2) an intermediate-dose CTV (commonly around 60 Gy equivalent) encompassing a 5 mm margin from the high-dose CTV, the entire NP, and high-risk anatomical subsites. This principle of “5 + 5” echoes the international consensus for head and neck squamous cell carcinoma [39], which is based on pathological studies of microscopic tumor infiltration in surgical specimens. Yet, the knowledge on microscopic spread in NPC is scarce.

In contrast, the Chinese protocol [40] recommends three dose levels for CTV_p_. According to this protocol, the high-dose CTV (approximately 66–76 Gy) encompasses the GTV_NP_ only. The intermediate-dose CTV (60–62 Gy) includes the GTV_NP_ with 5–10 mm margins and the entire NP, while the low-dose CTV (50–56 Gy) targets high-risk anatomical subsites. It is noteworthy that this protocol does not incorporate a margin to the GTV_NP_ to construct the high-risk CTV, as it is rooted in the belief that subclinical disease (i.e., CTV) has lower tumor density (<10^3^/mm^3^) compared to the GTV (>10^6^/mm^3^), and thus may not necessitate the full therapeutic dose. Consequently, it was shown in a dosimetric study that by eliminating the 5 mm CTV expansion in the 70 Gy volume, lower normal tissue complication probabilities (NTCP) in critical areas such as the brainstem, optic chiasm, lens, optic nerve, and parotid glands were observed [41]. Studies employing the Chinese protocol have reported promising outcomes. An updated analysis of 414 patients demonstrated a 5-year local control rate of 95% and regional relapse-free survival of 97%. However, it is important to note that in that cohort, 123 (29.7%) patients received a boost for residual disease (86/123 in the primary, and 37/123 in the cervical lymph node (LN)). Moreover, most patients (337/414, 81.4%) received IC and that the GTV_NP_ was defined with the pre-IC volume [42,43]. Nonetheless, the Chinese protocol was adopted in most of the recently published landmark trials [22,44,45,46,47] and no indications of inferior tumor control have been observed with this strategy.

Comparatively, the Chinese protocol exhibits two noteworthy characteristics: 1. the recommendation of a smaller overall volumes and lower dose levels [41,48]; 2. the allowance of hypo-fractionated and/or slightly accelerated boost dose to a limited volume around the GTV_NP_ [40]. It represents a viable strategy for volume reduction.

### 2.2. Redefining the Definition of High-Risk Anatomical Subsites

The current contouring guidelines, international [38] or Chinese [40], advocate for the prophylactic inclusion of various high-risk anatomical subsites. These encompass the bilateral parapharyngeal spaces (PPS), foramina ovale, foramina rotundum, foramina lacerum, petrous tips, pterygopalatine fossae (PPF), pterygoid fossae, and parts of the nasal cavities, maxillary sinuses, clivus, and sphenoid sinus, and in selected cases the cavernous sinus. The consideration of prophylactic irradiation of high-risk anatomical subsites emerged from the field borders used in 2D RT, where historically CT, endoscopy, and physical examination constituted the standard diagnostic tools. However, the advent of MRI and PET/CT has enabled radiation oncologists to visualize the extent of tumor infiltration with heightened precision. This has led to a growing debate regarding the necessity of including prophylactic anatomical subsites, and the appropriate extent.

Consequently, research groups have embarked on efforts to redefine this prophylactic volume, taking into account the laterality and stepwise spread of GTV_NP_.

#### 2.2.1. Unilateral NPC

Unilateral NPC, defined as a lesion confined to one side of the nasopharynx without crossing the midline, constitutes roughly 7% of all cases [49]. An investigation involving 176 cases of unilateral NPC revealed that the GTV_NP_ tends to invade adjacent tissues on the ipsilateral side [50].

In another classification by Wu et al. [51], NPC was considered central when the main body of the tumor was localized in the midline region of NP, and bilateral structures were symmetrically invaded. Other tumors that did not meet this definition were classified as eccentric. In their study of 870 MRIs, 72.4% were classified as eccentric, which was associated with low risk of concurrent bilateral tumor invasion (<10%). Notably, in eccentric tumors, the contralateral PPS, foramen lacerum, PPF and foramen ovale were infrequently involved, reported at 2.4%, 7.1%, 3.5%, and 0.2%, respectively.

These studies suggest that selected structures on the contralateral side may be excluded from the CTV in unilateral or eccentric NPCs.

#### 2.2.2. Stepwise Pattern of Spread

Another approach to defining high-risk anatomical subsites is grounded in the stepwise and continuous spread of tumors. Lin et al. [52] delineated two sets of CTV structures: (1) common structures highly susceptible to bilateral invasion and (2) downstream anatomical sites adjacent to involved areas along the routes of tumor infiltration. In their report of 220 patients, the 4-year local relapse-free survival rate was 94.7%, and the majority (10/11) had infield local recurrences.

In this landscape, Sanford et al. [53] retrospectively evaluated the treatment outcomes of the CTV delineation protocol at the Massachusetts General Hospital. Their approach was based on both tumor extent and the orderly stepwise pattern of tumor spread. Specifically, the CTV was determined by T-categories and tumor laterality. This small cohort, comprising 73 patients, reported a 5-year local control rate of 94%, with all local recurrences confined within the 70 Gy GTV target. However, it is important to recognize that this study was conducted in a non-endemic region, and proton beams were used in 84% of patients.

For early-stage tumors, a ten-year report [54] of 103 patients with T1-2 N0-1 NPCs treated using volume-reduced IMRT revealed only 1 case of local recurrence, which was classified as in-field. Notably, their protocol excluded several CTV structures in T1 tumors, including the lateral pterygoid muscles, post-styloid compartments, and the foramina ovale. It is noteworthy that the RT prescription in this cohort was accelerated and hypo-fractionated, consisting of 30 fractions with a permissible hotspot exceeding 107%.

Moving forward, Xie et al. [55] eliminated the prophylactic coverage of high-risk anatomical subsites in unilateral NPC cases. Their protocol described geometric expansions of 10 mm in the high-risk CTV and further 5–10 mm (+/− entire NP) in the low-risk CTV. After a median follow-up of 84 months of 95 patients, only 3 cases of local recurrence were observed, and all were within the PTV of the GTV_NP_.

It is essential to acknowledge that these diverse reports propose distinct strategies for individualizing CTV delineation, and a unified consensus remains elusive. Furthermore, no randomized trials have been conducted to establish the efficacy and safety of different volume reduction methods. Practically, many clinicians may find it prudent to incorporate a geometric expansion of the GTV_NP_ in the full therapeutic dose volume and include a prophylactic coverage of high-risk anatomical subsites to mitigate delineation uncertainties and interobserver variability [56,57].

### 2.3. Redefining the Elective Nodal Regions

NPC is characterized by bilateral lymphatic drainage, prompting guidelines to recommend prophylactic coverage of bilateral retropharyngeal (RP), II-V nodal levels [38,40]. Elective nodal levels were commonly covered with one (around 50 Gy equivalent) [40] or two dose levels (around 50 and 60 Gy equivalent) [38]. The pattern of nodal metastasis distribution has been extensively studied. The most commonly involved LN levels are II and RP [58,59], and skip metastasis in NPC is rare, occurring in only about 0.5% of cases [58]. Several landmark trials with randomized controlled and non-inferiority designs have contributed to the refinement of elective nodal volumes.

#### 2.3.1. Upper Neck Irradiation (UNI)

In a phase III trial [46], 446 patients with N0-1 disease were randomly assigned to receive elective UNI (i.e., levels II, III, and Va) or whole neck irradiation (WNI) (i.e., levels II-Vb) in the uninvolved neck (i.e., no cervical LN involvement) This study established that UNI is non-inferior to WNI in terms of 3-year regional relapse-free survival, at 97.7% and 96.3%, respectively. Moreover, the omission of level IV and Vb LNs reduced radiation doses to critical structures such as the thyroid, oesophagus, and trachea, leading to fewer late complications, including hypothyroidism, skin and neck tissue injury, and dysphagia. Of note, it was reported that the subgroup of patients who received unilateral UNI did receive significant scattered radiation to the lower neck as a result of the nine equally spaced coplanar fields, reporting a mean dose of 22 Gy [60]. This may be of relevance if a different beam arrangement or if proton beams are used.

In unilateral NPC, a retrospective study reported an 8% occurrence of contralateral lymph node metastasis. In patients without contralateral RP or level II involvement, less than 1% (1/104) displayed contralateral level III/Va metastases. This study suggested that prophylactic nodal coverage of contralateral RP and level II regions might suffice for unilateral NPC patients without contralateral LN metastasis, further enabling sparing of the level III [49].

#### 2.3.2. Submandibular (Level Ib) LN-Sparing

Level Ib involvement is seen in less than 5% of cases [58,59]. In the current guidelines, coverage of submandibular LN was recommended for high-risk patients. These high-risk factors include the involvement of structures draining to level Ib, a large size or the extra-nodal involvement (ENE) of level IIa LN and the involvement of ≥4 nodal regions in the ipsilateral neck [38,40]. Notably, recent reports suggest that omitting level 1b in selected high-risk patients may be safe. This includes cases with ENE and/or size >2 cm in level II [61,62,63] and/or ≥2 unilateral node-positive regions [62]. Pathological confirmation of radiological suspicious LNs has been advocated [63]. Nonetheless, it was reported that patients could still receive unintentional irradiation at the bilateral level Ib region with a mean dose of ≥50 Gy despite undergoing level Ib LN-sparing RT [62].

#### 2.3.3. Medial RP (Level VIIa) LN-Sparing

The RP LNs can be divided into medial and lateral groups. Medial RP LNs are primarily located at the C2-C3 levels [59], along or near the midline and medial to a line parallel to the lateral edge of the longus capiti muscle [64]. Of note, medial RP involvement is rare (0.3%) [59,65]. A randomized controlled trial [45] involving 568 patients demonstrated the non-inferiority of medial RPLN-sparing RT, with patients in the experimental arm achieving a 3-year local relapse-free survival of 95.3% compared to 95.5% in the control group. Importantly, none developed recurrence in the medial RP region. Sparing the medial RP LNs resulted in reduced volume and dose to the middle pharyngeal constrictor, glottic and supraglottic larynx, and reduced acute mucositis and dysphagia and late dysphagia.

#### 2.3.4. Retro-Styloid (VIIb) LN-Sparing

Consensus for elective coverage of the retro-styloid LN is low at 64% [38]. These nodes are the superior extension of level IIa with the cranial border at the skull base, which were involved in approximately 6.6% (178/2679) of NPC cases overall. However, most of them were found to be inseparable from the primary tumor or RP LN, and only 1.15% (31/2679) had isolated involvement of retro-styloid LN [66]. Omission of retro-styloid LN may be reasonable for tumors without posterior–lateral extension.

### 2.4. Redefining the Borders of Nodal Basins

The boundaries of nodal levels in NPC are primarily defined by the 2003 international consensus [67], later updated in 2013 [64]. However, these LN boundaries are not specific to the nodal distribution observed in NPC. This discrepancy has prompted several studies highlighting concerns and proposing refinements in nodal level definitions.

#### 2.4.1. Submandibular (Level Ib) Contouring

It was noted that level Ib LNs are scattered laterally and anteriorly to the submandibular gland [65]. Zhao et al. [68] further divided level Ib into four sublevels: intraglandular (IG), medial mandibular (MM), supra-perivascular (SP), and infra-perivascular (IP). Their analysis of 106 positive level Ib LNs from a cohort of 1518 patients revealed that 83% (88/106) and 16% (17/106) were involved at the SP and IP sublevels, respectively. Moreover, redefining prophylactic level Ib coverage to exclude the IG and MM sublevels resulted in reduced mean doses to the ipsilateral submandibular gland, bilateral sublingual glands, mandible, oral cavity, pharyngeal constrictor muscles, and supraglottic larynx.

#### 2.4.2. Level IIb Contouring

The current atlas defines the upper border of level IIb at the caudal edge of the lateral process of C1 [64]. However, NPC studies have demonstrated that up to 30% of level IIb nodes were located more cranial to this border [66,69]. Clinicians may need to consider adjusting the cranial border of level IIb, especially in cases where this level is involved.

#### 2.4.3. Level III/IV Contouring

A retrospective cohort reported that out of the 1184 and 350 cases of level III and level IVa involvement, only 2.8% and 0% were found anterior to the carotid sheath, respectively [66]. Moreover, another report [65] also indicated that no LN was observed in the gap between the sternocleidomastoid and infrahyoid ribbon muscles at level IVa. Refining the anterior border of these levels could potentially spare the thyroid and reduce late hypothyroidism.

#### 2.4.4. Level V Contouring

Many studies have suggested the potential oversight of the deep level V space [65,66,70,71]. The current atlas defines the posterior border of level V at the anterior border of the trapezius, and it does not describe the region posterior to it (i.e., the region between the trapezius muscle and the levator scapulae). The incidence of involvement in the deep level V space ranged from 1.3% to 5% [66,70,71]. Involvement of deep level V was associated with nodal disease at levels IVa [70], Va, Vb, and Vc [70,71], and it was observed in 13.3% of cases with level Vb LNs [65]. Consequently, adjustment of the posterior border of level Vb should be considered in selected high-risk patients [65,70].

Accumulating evidence suggests that tailoring the nodal target volume could optimize coverage while minimizing unnecessary radiation. However, it is vital to acknowledge the inherent limitations of observational and retrospective studies. This underscores the need for careful consideration before incorporating any changes into practice.

## 3. Dose and Volume Tailoring to Post-IC Volumes

Tailoring the RT dose and volume based on tumor response following IC holds potential for volume reduction. For locally advanced NPC, the current preferred treatment sequence involves IC followed by CCRT [12,72,73]. Approximately 2–11% of patients achieved a complete response (CR), while 77–86% had a partial response (PR) after IC. [9,10,21,47]. Furthermore, the reduction in volume of the primary tumor appeared to level off after two cycles of IC, whereas that of the affected LNs continued to decrease after the third cycle. A retrospective study documented volume reductions of 12.0%, 23%, and 20% in the NP tumor, 26%, 44%, and 42% in the RP LN, and 25%, 43%, and 55% in the cervical LN, following each successive cycle of IC [74].

Xiang et al. [75] reported the long-term outcome of 212 patients who were randomly assigned to receive RT using either pre-IC or post-IC volumes. In the post-IC arm, patients were administered 70 Gy in 33 fractions to the post-IC GTV_NP_ and 64 Gy in 33 fractions to the pre-IC GTV_NP_. With a median follow-up period of 98 months, the 5-year estimated survivals in the pre-IC and post-IC arms were comparable. Of note, the locoregional recurrence-free survival (LR-RFS) were 90.2% and 93.5% in the pre-IC and post-IC arms, respectively. All local recurrences in the post-IC arm were in-field. Importantly, patients who underwent volume reduction experienced fewer instances of xerostomia and hearing loss, along with an improved quality of life. However, it is worth noting that this study was conducted in a non-endemic region and predominantly included WHO type II NPC cases (79%).

In the endemic region, a phase II study [76] involving 112 patients implemented a treatment approach that delivered 68 Gy in 30 fractions to the post-IC volume and 60 Gy in 30 fractions to the pre-IC volume, following two cycles of IC. The study’s outcomes revealed remarkable 10-year LR-RFS, DMFS, and OS at 89.0%, 83.3%, and 75.9%, respectively. Notably, akin to the findings from Xiang’s study, all instances of local recurrence were in-field.

The contouring methods employed in the previous two reports had several similarities:

1. The pre-IC volume of GTV_NP_ was considered high-risk and was encompassed within the intermediate-dose CTV (treated with a dose of at least 60 Gy);
2. The pre-IC skull base or bony invasion were included within the post-IC volume and received the full prescription dose;
3. GTV of cervical LN was defined using the post-IC volume.

It is well documented that tumor response to IC, including tumor shrinkage and EBVDNA clearance, is prognostic for outcomes [77,78,79]. This response reflects the tumor’s biological behavior and inherent chemo-sensitivity, making it an important biomarker for treatment individualization. In addition, a retrospective study has suggested that IMRT alone may suffice in a subset of patients who achieved CR/PR after IC [80]. The omission of concurrent chemotherapy is currently being investigated in several phase III trials (ClinicalTrials.gov Identifiers: NCT05674305, NCT05527470, and NCT03015727).

Regarding RT, a single-arm phase II trial [81] treated low-risk stage III patients (defined as EBV DNA <4000 copies/mL) with two cycles of IC followed by 60 Gy CCRT for those who achieved CR or PR with an undetectable EBV DNA. This study showed promising 2-year PFS and locoregional relapse-free survival at 94% and 95%, respectively. However, this study was only published in abstract form after a median follow-up of 25.8 months. A full manuscript with updated analysis is eagerly awaited. Two ongoing randomized controlled trials are underway comparing reduced doses of either 60 Gy or 63.6 Gy in 30 fractions to 70 Gy in 33 fractions. Both studies recruited stage II/III patients who achieved CR or PR and EBVDNA clearance after IC (ClinicalTrials.gov Identifier: NCT04448522 and NCT05304468).

## 4. Dose and Volume Tailoring to Treatment Response during RT

Tailoring the RT dose and volume based on tumor response during the course of RT represents a potential window for treatment adaptation. Proactive adaptive radiotherapy (ART) represents scheduled replanning tailored to anatomical changes. Anatomical alterations are commonly encountered during RT, often attributed to weight loss and tumor shrinkage. Interestingly, some studies also observed the shrinkage of OARs, including the parotid and submandibular glands [82,83]. Furthermore, it has been demonstrated that changes in neck contour and set-up errors during RT can lead to a notable increase in the spinal cord and brainstem dose [84,85,86]. The introduction of replanning during the mid-course of RT using a new set of images may improve target coverage and better protect the normal tissues.

In a retrospective analysis of 290 patients who were enrolled in a prospective cohort [87], proactive replanning at the 15th and/or 25th fraction was performed for half of the patients, while the other half declined. The replanning group demonstrated a higher 8-year LR-RFS rate of 87.4% compared to 75.6% in the non-replanning group, despite no significant improvement in OS. These patients also reported less dry mouth and sticky saliva. However, the effectiveness of ART in reducing xerostomia is debatable. The ARTIX trial [88] randomized patients with locally advanced oropharyngeal squamous cell carcinoma to weekly replanning but failed to show a reduction in xerostomia in terms of stimulating salivary flow by paraffin.

Furthermore, the appropriate timing for proactive replanning remains unclear. Weekly kilovoltage cone-beam CT scans of 13 patients revealed that 11 cases (84.6%) experienced ≥50% shrinkage of GTV before the 21st fraction, which increased to 12 cases (92.3%) before the 26th fraction [82]. Another study suggested two replans at the 5th and 15th fractions after assessing anatomic and dosimetric changes of target volume and OARs [89].

However, the optimal method to adapt target volumes remains to be defined. Some clinicians adjust treatment volumes based solely on anatomical changes, while others advocate shrinking the high-dose volume to the residual tumor. In this context, a two-phase technique has been described [90,91,92]. In a report by Xie et al., the phase I delivered doses of 53–54 Gy, 47.5 Gy, and 45 Gy to the GTV, high-risk CTV and low-risk CTV, respectively, over 25 fractions. In phase II, doses of 15–15.5 Gy and 13.5 Gy were delivered to the residual GTV and high-risk CTV, respectively, over seven fractions. Of note, the GTV was adapted in phase II while the high-risk CTV remains unchanged, ensuring that the regressed tumor receives a total dose of at least 65 Gy. Preliminary results indicated a local recurrence-free survival of 90.5%, with no recurrence observed in the regressed area [90]. Another emerging approach involves a mixed-beam arrangement [91], in which the first IMRT phase targeted both the high- and low-risk CTVs, followed by a proton phase for the high-risk regions. This approach allows the proton therapy to target the upper neck, mitigating uncertainties linked to tissue inhomogeneity stemming from tumor shrinkage and positioning errors that are often encountered in the later stages of RT.

ART is conventionally time and labor-intensive. Implementing ART necessitates meticulous technological considerations on image quality, deformable image registration and dose accumulation [93]. Many studies had focused on predicting or selecting patients who may benefit the most from ART, and currently, ART for head and neck cancer (HNC) is predominantly offline and ad hoc [94]. However, online daily ART for HNC is gaining momentum with technologies such as MRI-LINAC and the Varian Ethos^TM^ system, and the integration of artificial intelligence for auto-segmentation [95,96] and re-optimization (e.g., RapidPlan). However, whether intensive adaptive planning would translate into clinical benefits in terms of improved tumor control and reduced toxicity remains to be determined. More studies akin to ARTIX are eagerly anticipated for NPC.

## 5. Dose Escalation Tailored to Biological Imaging

Locoregional failures observed in NPC are predominantly infield, which has prompted the exploration of dose escalation strategies to enhance local control. Data from the conventional RT era had suggested that a boost dose was correlated with enhanced local control [97]. However, achieving dose escalation across the entire tumor while minimizing adjacent normal tissue toxicity is challenging. Target volume definitions have traditionally relied on anatomical volume. However, the emergence of biological imaging, which provides insights into the metabolic, biochemical, physiological, functional, molecular, genotypic, and phenotypic characteristics of tumors, has introduced a valuable tool for delineating functionally active or potentially radioresistant sub-volumes within tumors, referred to as the biological target volume (BTV) [98]. This approach allows for customized dose delivery.

### 5.1. ^18^F-Fluorodeoxyglucose (^18^F-FDG) PET-CT

^18^F-FDG-PET-CT is a molecular imaging technique reflecting cancer metabolism. Various thresholds have been proposed for tumor volume definition [99,100,101]. A retrospective comparative study involving 292 patients who underwent PET-guided RT employed three distinct criteria for defining the GTV. In Group 1, visual criteria were used; Group 2 utilized a standardized uptake value (SUV) threshold of 2.5, while Group 3 employed the visual criteria for GTV, and defined a sub-volume (named GTV-PET) using threshold of 50% of the maximal SUV. Dose prescription for the GTV ranged from 70.4 to 72.6 Gy in 32 to 33 fractions. Additionally, the GTV-PET in Group 3 received simultaneous integrated boost of 75.2 to 77.55 Gy in 32 to 33 fractions. The results revealed that dose-painting in Group 3 correlated with improved 5-year local and distant recurrence-free survival and OS, without additional G3-G4 toxicities [99].

### 5.2. ^18^F-Fluoromisonidazole (FMISO) PET-CT

Intra-tumoral hypoxia is believed to contribute to radio-resistance, which dose escalation can potentially overcome. ^18^F-FMISO, a nitroimidazole derivative, accumulates in hypoxic viable cells but not necrotic cells [102]. A feasibility study utilized ^18^F-FMISO PET-CT to deliver a boost dose of 14 Gy (to a total of 84 Gy) to the hypoxic sub-volume (defined as a tumor–muscle ratio > 1.3) while respecting the conventional OAR constraints. It was shown to be achievable using both IMRT and volumetric-modulated arc therapy (VMAT) techniques [103]. A proton-based planning study explored the feasibility of delivering a stereotactic boost of 10 GyE in two fractions to an FMISO PET-defined hypoxic sub-volume before the course of standard 70 GyE radiation. However, in their cohort, 3 out of 8 patients failed to meet the constraint in the temporal lobe [104].

### 5.3. Diffusion-Weighted MRI (DWI)

The utility of DWI in defining boost volume was investigated based on the theory that viable parts of a tumor exhibit restricted diffusion and lower apparent diffusion coefficient (ADC) compared to necrotic parts [105]. In a randomized study of 260 locally advanced NPC cases, the dose-painting group received doses of 75.2 to 77.55 Gy in 32–33 fractions to parts of tumor with ADC below the mean ADC, according to the pre-IC MRI. As compared to the control group receiving conventional 70.4–72.6 Gy in 32–33 fractions, the dose-painting group demonstrated improved 2-year disease-free survival, local recurrence-free survival, distant metastasis-free survival (DMFS), and OS. No additional grade 3 or above acute or late adverse events were observed [106].

With the increasing availability of integrated ^18^F-FDG PET and MR (PET/MR) scanners, a pilot study demonstrated that volumes defined by DWI and PET did not completely overlap. More than 90% of the volume of interest (VOI) defined by DWI was enclosed in the PET-defined VOI (defined as SUVmax > 40%), while only around half of the PET-defined VOI was encompassed in the DWI-defined VOI [107]. The findings suggested that PET and DWI may complement each other in defining the optimal sub-volume for dose escalation.

All in all, preliminary findings indicate that dose-painting holds promise for improving local tumor control. However, lessons learnt from other HNC underscored the potential late complications of dose escalation such as mucosal ulcers and dysphagia [108,109]. The importance of long-term safety data cannot be overstated. Moreover, prospective data are needed to assess the comparative efficacy and safety of different dose-painting strategies and to identify high-risk patients who could benefit from dose escalation. In addition to the conventional clinicopathologic features, radiomics [110] and genomics [111,112] hold potential in predicting radio-resistance and selecting suitable patients for dose-painting.

## 6. Dynamic Decision Making Guided by EBVDNA

Non-keratinizing NPC is consistently associated with EBV infection [113] and the EBV in episomal forms released into the peripheral circulation upon tumor lysis. To date, most data on plasma EBVDNA predominantly utilized real-time qPCR that targets the BamHI-W repeat region of the EBV genome [114]. Plasma EBVDNA has emerged as an important biomarker implicated in NPC screening [115], treatment [116,117], and surveillance [117,118]. Furthermore, EBVDNA levels are dynamic during treatment and demonstrate prognostic significance at various time-points, leading to their increasing integration into clinical trials for patient selection and treatment adaptation. However, challenges in harmonizing assays have hindered knowledge generalization [119].

### 6.1. Pre-Treatment EBVDNA

Elevated pre-treatment levels of EBVDNA are indicative of a less favorable prognosis [116,120]. It has had implications for the consideration of induction, concurrent, or adjuvant chemotherapy in recent pivotal trials. A phase II non-inferiority randomized controlled trial suggested that two cycles of concurrent cisplatin (at 100 mg/m^2^) were non-inferior to three cycles in low-risk stage III-IVb patients with pre-treatment EBV DNA of <4000 copies/mL. The 3-year PFS was 88% for the two-cycle group and 90.4% for the three-cycle group, resulting in a difference of 2.4% (95% CI: −4.3 to 9.1). The result met the predefined non-inferiority margin of 10%. Notably, patients in the three-cycle group experienced significantly higher acute toxicity burden and late adverse events [121]. In addition, Zhang et al. [11] demonstrated that induction gemcitabine–cisplatin improved 5-year OS only in the subgroup with pre-treatment EBV DNA ≥ 4000 copies/mL. In a randomized study [22] involving stage II and T3N0 NPC patients without adverse features, RT alone was shown to be non-inferior to CCRT in terms of 3-year failure-free survival. In this study, EBV DNA levels ≥ 4000 copies/mL were identified as one of the adverse features leading to patient exclusion. In the adjuvant setting, Miao et al. [15] enrolled high-risk patients to receive adjuvant capecitabine, including those harboring pre-treatment EBV DNA levels > 20,000 copies/mL.

### 6.2. Post-IC EBVDNA

A subsequent window for risk stratification and treatment adaptation emerges following IC. Patients achieving EBVDNA clearance after IC exhibit a more favorable prognosis compared to those without [77]. Results of ongoing trials studying reduced-dose RT for patients with EBVDNA clearance after IC are eagerly awaited (ClinicalTrials.gov Identifier: NCT04448522 and NCT05304468).

### 6.3. Post-RT EBVDNA

Post-RT EBVDNA is the most adverse prognostic factors among other predictors including pre-treatment EBVDNA, and the T/N-category [116]. It was postulated that adjuvant chemotherapy can eliminate residual tumor clones after RT, and the presence of which could be reflected in the post-treatment EBVDNA level. In the NPC-0502 study [122], Chan et al. recruited patients with positive EBVDNA levels 6–8 weeks after RT to receive adjuvant chemotherapy. Importantly, they discovered that approximately 30% of these patients who had positive EBVDNA either demonstrated persistent or metastatic disease upon re-staging. However, this study failed to show clinical benefit of adjuvant gemcitabine–cisplatin. It was postulated that the lack of benefit could be due to the late commencement of adjuvant chemotherapy at a median of 13 weeks post-RT, and the selection of patients with extremely high risk of recurrence. Moving forward, many ongoing trials have incorporated post-treatment EBVDNA assessment to identify high-risk patients to receive adjuvant chemotherapy and/or immune check-point inhibitors (ICPi), for example, the NRG-HN001 ClinicalTrials.gov Identifier: NCT02135042, and NCT05517135.

## 7. RT in the Era of Immunotherapy

ICPi targeting programmed death receptor 1 (PD-1) or its ligand (PDL1) or cytotoxic T-cell lymphocyte-associated protein 4 (CTLA-4) have shown effectiveness in treating recurrent/metastatic NPC [123,124,125,126]. Ongoing research is now exploring the role of ICPi in definitive treatment. Phase II single-arm trials for pembrolizumab [127] and tislelizumab [128] (both PD-1 inhibitors) have studied the efficacy of integrating PD-1 inhibitors with IC and CCRT, followed by maintenance therapy, with results presented in abstract form. The phase III CONTINUUM [129] study recruited patients with stage III-IVA NPC (except T3/T4N0 or T3N1). The experimental arm received up to 12 cycles of induction, concurrent and maintenance sintilimab (a PD-1 inhibitor), and it demonstrated improved 3-year event-free survival compared to the control arm treated with IC +CCRT (86.1% vs. 76%; stratified HR 0.59). The full manuscript of this study is eagerly anticipated. Moving forward, the TIRA [130] trial evaluated treatment deintensification by omitting concurrent cisplatin. In the experimental arm, patients received IC and induction–concurrent–maintenance toripalimab (a PD-1 inhibitor).

In the era of immunotherapy, there is a pressing need to identify biomarkers for PD1/PDL1 therapy in NPC. Conventional markers like PDL1 expression and tumor mutation burden have not demonstrated strong predictive significance in NPC [123,124,131]. The CONTINUUM [129] study proposed that the clinical benefit of sintilimab was observed only in patients with tertiary lymphoid structure (TLS), the ectopic lymphoid tissues that can be found in the tumor or neighboring peripheral tissue [132]. Genomic studies on the tumor microenvironment may offer further insights [124,133]. Chen et al. studied the gene expression patterns in NPC and identified three immune subtypes: active, evaded, and non-immune. They found that patients with an active immune subtype responded better to ICPi [133]. Further research is needed to define this landscape.

The interaction between RT and immunotherapy has become a prominent subject of research in solid tumors, including HNC. Preclinical data suggest that radiation can have immunostimulatory effects, for example, by triggering immunogenic cell death, enriching the immune tumor microenvironment, and overexpressing MHC class-I and Fas receptors on tumor to activate T cell response [134,135]. This RT-induced immune response is thought to synergize with immunotherapy. Yet, the optimal combination of RT and immunotherapy in terms of dose, volume, schedule, and sequence is still under investigation.

The lack of clinical benefit of ICPi in the definitive treatment of HNC [136] has led to the postulations that elective nodal treatment inhibits the priming of T-cells naturally harbored in the lymph node chains, thus dampening tumor immune response. This phenomenon has been observed in mouse models [137,138]. Consequently, a strategy has been developed based on preclinical models for HNC, which involves delivering stereotactic body RT to the primary tumor in combination with immunotherapy, followed by delayed nodal treatment. Their analysis suggests that this lymph node-sparing approach can induce a systemic immune response and produce anti-tumor responses at local, regional, and distant sites [138]. In the context of NPC, the recent movement towards volume-reduced RT, as discussed in previous sections, in particular studies focusing on limited neck irradiation [46], may offer a potential avenue for optimizing the combination of immunotherapy and RT. However, further research is essential to fully understand how RT interacts with immunotherapy in NPC and to determine the optimal treatment strategy.

## 8. Conclusions

Long accustomed to established practices and guidelines, oncologists have traditionally employed a one-size-fits-all approach in RT planning, and emphasized treatment efficacy over potential long-term complications in managing this highly curable cancer. However, as the landscape in NPC evolves, volume-reduced RT with refined target volumes and adaptive planning has demonstrated safety and efficacy. Ongoing research is actively exploring further risk-adapted strategies that incorporate functional imaging and biomarkers to enhance treatment efficacy and minimize toxicity. Beyond the subjects addressed in this review, continued research focused on multi-omics offers the potential to provide deeper insights into NPC. The collaborative efforts to explore and validate these individualized strategies reflect a dedication to advancing NPC treatment and improving the quality of life of NPC survivors. 

## Figures and Tables

**Table 1:** Individualized treatment strategies in NPC.

	Areas of Interest
Tools for risk stratification	Clinicopathologic features
	Biological imaging, e.g., 18F-fluorodeoxyglucose PET-CT, 18F-fluoromisonidazole PET-CT, and diffusion-weighted MRI
	Biomarkers, e.g., EBV DNA
	Other multi-omics, e.g., radiomics and genomics
Timing of treatment adaptation	Pre-treatment
	Post-induction chemotherapy
	During RT
	Post-RT
Systemic therapy	Induction, concurrent and adjuvant chemotherapy – Selection criteria, dose, and regimen
	Immune checkpoint inhibitors – Selection criteria, induction, concurrent and maintenance regimen
Radiotherapy	Volume reduction 1. Redefining dose and margins of CTV_primary_ 2. Redefining high-risk anatomical subsites – Omission, unilateral NPC, stepwise pattern of spread 3. Redefining elective nodal regions – Upper neck irradiation, submandibular lymph node sparing, medial retropharyngeal lymph node sparing, retro-styloid lymph node sparing 4. Refining the borders of nodal basins 5. Dose and volume tailoring to post-induction chemotherapy volumes
	Dose adaptation 1. Risk-adapted dose de-escalation tailoring to the response of induction chemotherapy 2. Dose-painting or dose escalation with biological imaging
	Adaptive RT – Dose and volume tailoring to treatment response during RT

Abbreviations: CTV, Clinical target volume; EBV, Epstein–Barr virus; NPC, Nasopharyngeal carcinoma; RT, Radiotherapy.
